# Metabolomics for the Diagnosis of Secondary Infections in Critically Ill Patients With COVID-19

**DOI:** 10.1097/CCE.0000000000001336

**Published:** 2025-11-06

**Authors:** Gordan McCreath, Clément Regnault, Gavin J. Blackburn, Rónán Daly, Alistair T. Leanord, Phillip D. Whitfield, Andrew J. Roe, Alan Davidson, Malcolm J. Watson, Malcolm A. B. Sim

**Affiliations:** 1 School of Infection and Immunity, College of Medical, Veterinary, and Life Sciences, University of Glasgow, Glasgow, Scotland.; 2 College of Medical, Veterinary and Life Sciences Shared Research Facilities, University of Glasgow, Glasgow, Scotland.; 3 School of Medicine, Dentistry and Nursing, College of Medical, Veterinary and Life Sciences, University of Glasgow, Glasgow, Scotland.

**Keywords:** biomarkers, coinfection, COVID-19, critical illness, metabolomics

## Abstract

**OBJECTIVES:**

Secondary infections are a common occurrence in critically ill COVID-19 patients. These are difficult to identify, and antibiotic usage is high in this population. Identification of biomarkers for secondary infections would help to ensure antibiotics are being utilized only for patients who require them. This study sought to identify a panel of biomarkers capable of distinguishing critically ill COVID-19 patients with and without secondary infections.

**DESIGN:**

A multicenter retrospective cohort study.

**SETTING:**

Three critical care units in Scotland, United Kingdom.

**PATIENTS:**

One hundred five patients admitted to critical care with COVID-19, and 49 healthy volunteer controls.

**INTERVENTIONS:**

None.

**MEASUREMENTS AND MAIN RESULTS:**

Serial blood samples were obtained from critically ill COVID-19 patients with and without confirmed secondary infections, and a single sample was collected from healthy volunteers to provide baseline metabolic profiles. Metabolomic analysis was performed using liquid chromatography-mass spectrometry, and metabolites that were significantly different between patients with and without secondary infections were identified. Additionally, metabolites capable of distinguishing Gram-positive from Gram-negative organisms were also investigated. Forty patients developed a secondary infection during the study period. A significant increase in metabolites creatine and 2-hydroxyisovalerylcarnitine, and a significant reduction in S-methyl-L-cysteine were detected in patients with secondary infections. This metabolite panel could identify patients with secondary infections with an area under the curve (AUC) of 0.83 (95% CI, 0.68–0.97). Metabolites differentiating Gram-positive and Gram-negative infections included betaine, N(6)-methyllysine, and phosphatidylcholines (PCs; 38:6), PC(38:4), PC(40:6), and PC(36:4) with an AUC of 0.88 (95% CI, 0.68–1.0).

**CONCLUSIONS:**

Metabolomic profiling of critically ill COVID-19 shows promise for identification of novel biomarkers for secondary infections. Larger validation studies will help to confirm these findings.

**KEY POINTS**

**Question**: Can metabolomics identify biomarkers capable of identifying secondary infections in critically ill COVID-19 patients?

**Findings**: A panel of three metabolites could successfully differentiate COVID-19 patients with a secondary infection from those without. An additional panel of six metabolites could distinguish Gram-positive and Gram-negative infections.

**Meaning**: Metabolomics can identify metabolite biomarkers in critically ill COVID-19 patients with secondary infections, which may aid in future diagnosis.

The illness COVID-19 caused a global pandemic and worldwide health emergency of unprecedented proportion. Since the outbreak began at the end of 2019, more than 770 million COVID-19 cases have been recorded, with approximately 7 million deaths being attributed to the illness (1). High rates of immunity from vaccination and prior infection has led to a marked reduction in the severity of COVID-19; however, a subset of patients may still progress to severe illness characterized by critical hypoxia requiring invasive mechanical ventilation (IMV) (2). Patients who are elderly, obese, pregnant, or those with comorbidities such as diabetes, hypertension, and active cancer are particularly at risk of severe illness (3, 4). Furthermore, viral variants with increased transmissibility capable of immune escape have emerged, leading to vaccine breakthrough infections (5). As a result, COVID-19 may continue to pose an ongoing burden to critical care units.

A potential complication of COVID-19 is the development of a secondary infection. A meta-analysis by Calderon et al (6) found that secondary infections are uncommon in the overall cohort of patients hospitalized with COVID-19, with a rate of 12%. However, the rate is considerably higher in critically ill patients and is associated with a longer ICU length of stay and an increased mortality rate (6, 7). Alarmingly, a much greater proportion of critically ill patients will receive antibiotics as part of their treatment strategy, with 86% of critically ill COVID-19 patients receiving antimicrobial therapy (6).

Severe COVID-19 and bacterial sepsis share many of the same inflammatory pathways (8). Biomarkers for bacterial sepsis overlap with those used for severe COVID-19, such as leukocyte count, C-reactive protein (CRP), and procalcitonin (9). Furthermore, patients with severe COVID-19 exhibit many of the same clinical manifestations as those with bacterial sepsis (10). Diagnostic scoring systems such as the confusion, urea, respiratory rate, blood pressure, and age above or below 65 years and quick Sequential (Sepsis-Related) Organ Failure Assessment scores have been shown to perform poorly in patients with COVID-19 (11). Thus, identifying COVID-19 patients with secondary infections presents a diagnostic challenge. Culture and sensitivity represent the gold standard for identification of infection, but this is a time-consuming and laborious process. Additionally, false positives are frequently seen, particularly if samples are taken after antibiotics have been administered (12). A lack of rapid diagnostic tools for secondary infections necessitates empirical treatment, often with broad-spectrum antibiotics, which can carry significant side effect profiles and increase proliferation of multidrug-resistant organisms.

The systemic inflammatory response seen in sepsis results in major dysregulation of host metabolic pathways. As a result, the metabotype, or metabolic phenotype of patients, can become significantly deranged (13). Metabolomics is the use of spectroscopic technology to detect and quantify metabolites (molecules < 1000 kDa) in various biofluids. This can provide a snapshot of the metabolic status of an individual, and can, therefore, be used as an indicator of cellular health at a given point in time (14). Pathologic changes in cellular metabolic processes are reflected in alterations to the serum metabolome. Detection and quantification of changes in metabolite concentration may be indicative of patients with secondary infections in COVID-19 and aid in biomarker discovery. Multiple studies have sought to utilize metabolomics to identify biomarkers of sepsis; however, studies examining biomarkers for secondary infections in COVID-19 are lacking. In this study, we sought to elucidate the metabolomic profile of adult patients admitted to critical care with COVID-19 to identify biomarkers capable of diagnosing secondary infections.

## MATERIALS AND METHODS

### Study Design and Participants

This was a prospective, diagnostic, observational study conducted in the high-dependency unit (HDU) and ICU of three Scottish Hospitals (the Queen Elizabeth University Hospital, Glasgow; Glasgow Royal Infirmary, Glasgow; and the Royal Alexandra Hospital, Paisley) between November 2020 and October 2021. Ethical approval was gained from the Scotland Research Ethics Committee on the August 26, 2020 (Title: The Diagnostic Use of Metabolomics for the Early Recognition of Sepsis, reference number 17/SS/0062). Patients within HDU required level 2 care, defined as those in need of enhanced observation or intervention beyond standard ward-level care to prevent clinical deterioration. This included individuals requiring continuous monitoring and support for two or more basic organ systems, or advanced support for a single organ system, excluding respiratory support. Patients in ICU received level 3 care, defined as requiring mechanical ventilation and/or advanced support for multiple organ systems including vasopressor administration and renal replacement therapy (15). Patient recruitment occurred in adherence to the Declaration of Helsinki. Patients 18 years old and over admitted to a critical care unit who had tested positive for severe acute respiratory syndrome coronavirus 2 (SARS-CoV-2) in the previous 7 days were eligible for recruitment. Pregnant patients and those who were not expected to survive their illness were excluded from enrollment. A control group of healthy volunteers was also recruited who served to provide a normal baseline metabolic profile. This group consisted of individuals with no past medical history and who did not take any regular medication, had not had a previous SARS-CoV-2 infection, and had not yet been vaccinated against SARS-CoV-2. A secondary infection was deemed to be present if a patient had a positive microbiological culture and met the criteria for one of the definitions of infection adapted from the Centers for Disease Control and Prevention/National Healthcare Safety Network definitions (16) (**Supplementary Table 1**, https://links.lww.com/CCX/B570). Where there was diagnostic uncertainty, opinion was sought from a consultant clinical microbiologist who acted as an independent adjudicator.

Blood samples were gathered from patients at the time of recruitment (day 0), and subsequent samples were collected on days 3 and 10. Critically ill patients who showed evidence of a secondary infection from positive microbiological cultures or clinical deterioration attributed to infection had an additional blood sample taken at that time point, providing a potential maximum of four blood samples per patient. Routine laboratory and clinical data were gathered daily for each patient during the trial period.

Samples were left to clot for 1 hour before centrifuging at 2000*g* for 15 minutes. Serum was aliquoted and stored at –80°C. Metabolite extraction was performed by adding 25 μL of defrosted serum to 1 mL of chloroform:methanol:water solvent in a 1:3:1 ratio (v/v/v) before centrifuging for 3 minutes at 13,000*g* and collecting a 200 μL aliquot for metabolomic analysis.

### Metabolomic Analysis

Untargeted metabolomic analysis was performed via liquid chromatography-mass spectrometry in both positive and negative ionization modes with the Thermo Orbitrap QExactive system (Thermo Fisher Scientific, Bremen, Germany) in conjunction with a Dionex UltiMate 3000 Rapid Separation LC platform (Thermo Fisher Scientific, Hemel Hempstead, United Kingdom) with a zwitterionic polymeric hydrophilic interaction chromatography column and ammonium carbonate in water/acetonitrile gradient. Data were processed using the XCMS (Leibniz Institute of Plant Biochemistry, Halle, Germany) (17) and MZMatch (Groningen Biomolecular Sciences and Biotechnology Institute, University of Groningen, Groningen, The Netherlands) (18) computational tools. Putative metabolite identification was performed through comparison of mass to charge ratios (*m/z*) of peaks with database values. Metabolite identities were confirmed by matching *m/z* and retention times to known authentic standards, and/or by tandem mass spectrometry comparing fragmentation spectra with those of authentic standards or compounds in databases.

### Statistical Analysis

Principal component analysis (PCA) was initially used to look for clustering of data and to detect confounders. The R limma package (The Walter and Eliza Hall Institute of Medical Research, Parkville, VIC, Australia) was then utilized to identify peaks that differed between groups (19). The resulting *p* values were corrected for multiple comparisons using the R q-value package (Princeton University, Princeton, NJ), which were chosen post hoc (20). Bayesian logistic regression classifiers were constructed to predict whether a given sample had a secondary infection and to distinguish Gram-positive and Gram-negative infections. These used the R caret (21) (Pfizer Global R&D, Groton, CT) and arm (Columbia University, New York, NY) (22) packages and used a ten-fold cross-validation procedure, repeated ten times to gauge validated performance. Dot plots were used to illustrate significantly different metabolites, and receiver operating characteristic (ROC) curves were produced to show test performance via the area under the curves (AUCs) and 95% CIs.

## RESULTS

### Patient Data

**Figure 1.**
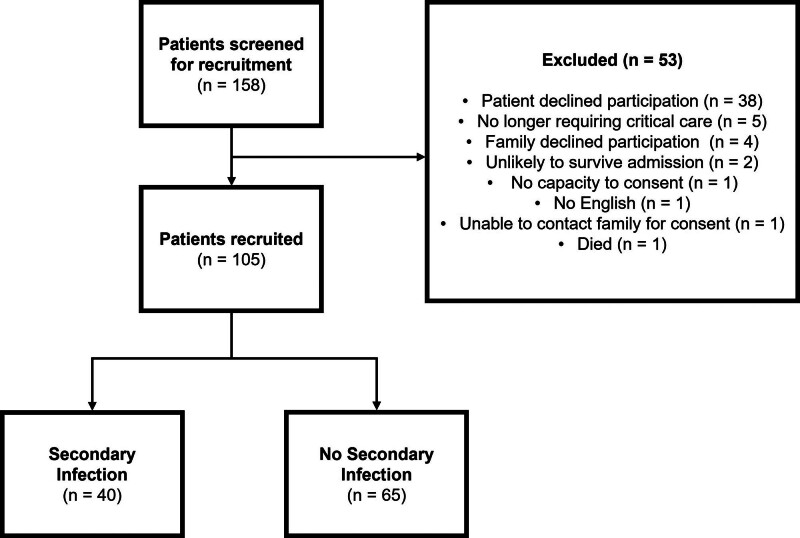
Patient selection diagram.

Between November 2020 and October 2021, a total of 105 patients were recruited to the study. Additionally, 49 healthy volunteers also agreed to participate. **Figure 1** outlines the patient recruitment.

**Table 1** provides the background demographics and clinical data. During the study period, 40 of 105 critically ill patients (38.1%) developed a secondary infection (**Supplementary Table 2**, https://links.lww.com/CCX/B570). A greater number of males were recruited; however, secondary infections were more frequent in female patients. The most common comorbidities were hypertension, diabetes, and asthma. Patients with secondary infections had both a longer critical care and hospital stay than those without. A larger proportion of patients with secondary infections died (42.5%) compared with those without secondary infections (29.2%). Patients requiring IMV frequently developed secondary infections, with 92.5% of secondary infection cases occurring in ventilated patients.

**TABLE 1.** Comparison of Demographics and Clinical Data Between Patients With and Without Secondary Infections

Characteristics	Secondary Infection, *n* = 40 (38.1%)	No Secondary Infection, *n* = 65 (61.9%)
Age, yr, median ± IQR	59 ± 13.75	57 ± 11
Sex, *n* (%)		
Female	22 (55)	22 (33.8)
Male	18 (45)	43 (66.2)
Ethnicity, *n* (%)		
White	34 (85)	55 (84.6)
Asian, Asian Scottish, or Asian British	3 (7.5)	4 (6.2)
Black, Caribbean, or African	1 (2.5)	3 (4.6)
Multiple ethnicities	1 (2.5)	0 (0)
Other ethnic group	1 (2.5)	0 (0)
Not recorded	0 (0)	3 (4.6)
Body mass index, median ± IQR	32 ± 8.15	32 ± 9.1
Days from severe acute respiratory syndrome coronavirus 2 infection to hospital admission, median ± IQR	5 ± 7.25	3 ± 6.5
Days from hospital admission to critical care admission, median ± IQR	1 ± 2.25	1 ± 2
Length of ICU stay, median ± IQR	18 ± 15	8 ± 12.5
Length of hospital stay, median ± IQR	29 ± 30.5	13 ± 17
Outcome, *n* (%)		
Survived	23 (57.5)	46 (70.8)
Died	17 (42.5)	19 (29.2)
Invasive mechanical ventilation, *n* (%)	37 (92.5)	23 (35.4)
Renal replacement therapy, *n* (%)	4 (10)	7 (10.8)
Comorbidities, *n* (%)		
Hypertension	13 (32.5)	18 (27.7)
Diabetes mellitus	9 (22.5)	11 (16.9)
Asthma	10 (25)	8 (12.3)
Immunosuppression	9 (22.5)	9 (13.8)
Ischemic heart disease	3 (7.5)	4 (6.15)
Active cancer	2 (5)	5 (7.7)
Chronic obstructive pulmonary disease	1 (2.5)	5 (7.7)

IQR = interquartile range.

### Metabolites Identifying Secondary Infections

**Figure 2.**
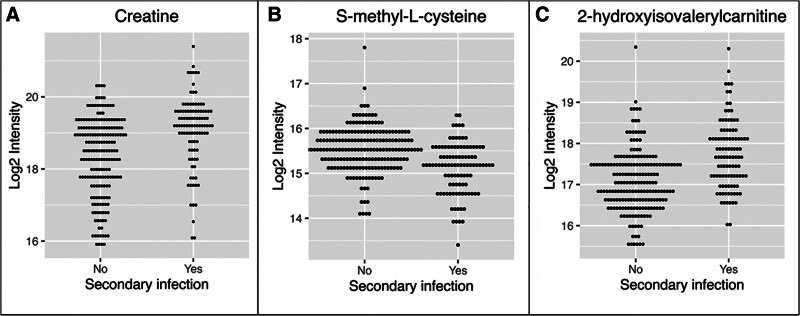
*Dot plots* depicting significantly different metabolites with and without secondary infections. **A**, Creatine. **B**, S-methyl-L-cysteine. **C**, 2-hydroxyisovalerylcarnitine.

PCA was performed on samples from patients with and without secondary infections, as well as healthy volunteer samples (**Supplementary Fig. 1**, https://links.lww.com/CCX/B570). This aided in filtering of background signals and correcting for covariates such as extraction date, location, sedation, and paralysis use. Initial metabolomic analysis yielded 32 peaks that were significantly different between patients with and without secondary infection. After exclusion of poor-quality signals, isotope peaks, and nonannotated peaks, five peaks were identified corresponding to three separate metabolites (**Fig. 2**; **Table 2**; and **Supplementary Figs. 2–6**, https://links.lww.com/CCX/B570). The metabolites creatine and 2-hydroxyisovalerylcarnitine were significantly increased in secondary infections, while S-methyl-L-cysteine was significantly reduced.

**TABLE 2.** Metabolites Significantly Different Between Patients With and Without Secondary Infections

Metabolite	Direct Parent	Molecular Formula	Mass to Charge Ratio	Retention Time (s)	Relative Intensity (Mean ± se)		Log2 Fold Change	q-Value
					Secondary Infection	No Secondary Infection		
Creatine	Alpha amino acids and derivatives	C_4_H_9_N_3_o_2_	130.0622	724	19.075 ± 0.0068	18.348 ± 0.0775	0.056	0.0573
S-methyl-L-cysteine	Cysteine and derivatives	C_4_H_9_No_2_S	134.0282	623	15.119 ± 0.004	15.541 ± 0.036	–0.04	0.0573
2-hydroxyisovalerylcarnitine	Acyl carnitines	C_12_H_23_No_5_	262.1647	541	17.734 ± 0.0059	17.069 ± 0.0535	0.055	0.0859

A q-value < 0.1 indicates statistical significance.

**Figure 3.**
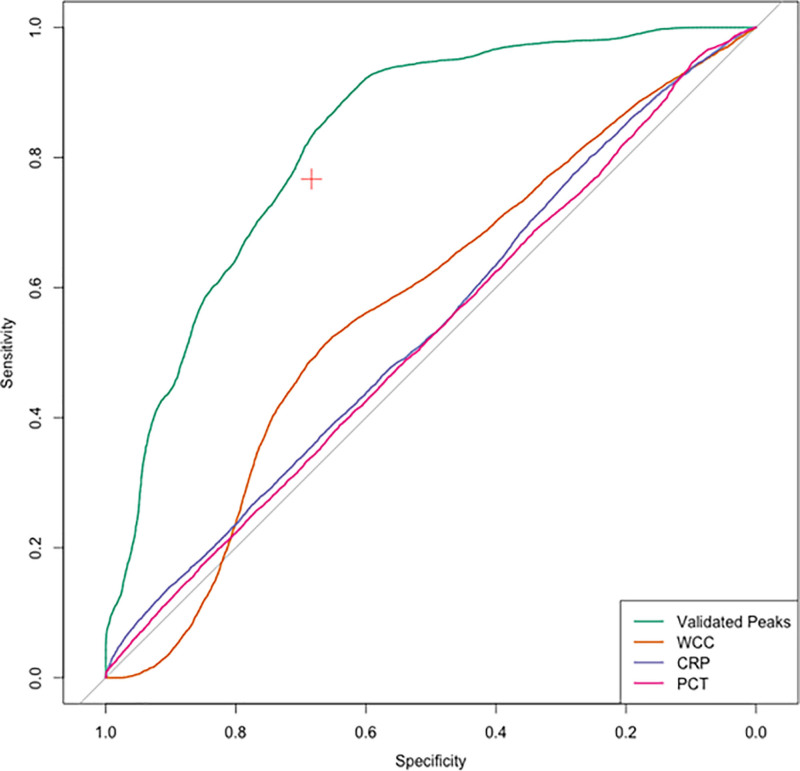
Receiver operating characteristic curves demonstrating test performance for identifying secondary infections. *Curves* represent the metabolomic panel comprising the three validated peaks: creatine, S-methyl-L-cysteine, and 2-hydroxyisovalerylcarnitine, as well as inflammatory markers, including total white cell count (WCC), C-reactive protein (CRP), and procalcitonin (PCT). *Red plus* represents sensitivity and specificity of clinician diagnosis of secondary infection based on antibiotic usage.

**Figure 3** shows the ROC curves depicting the sensitivity and specificity of the metabolomic panel for diagnosis of secondary infections compared with traditional markers of infection. The biomarker panel demonstrated superior test performance (AUC, 0.83; 95% CI, 0.68–0.97) compared with total white cell count (WCC: AUC, 0.59; 95% CI, 0.37–0.80), CRP (AUC, 0.48; 95% CI, 0.25–0.70), and procalcitonin (AUC, 0.50; 95% CI, 0.28–0.72).

### Metabolites Differentiating Gram-Positive and Gram-Negative Infections

**Figure 4.**
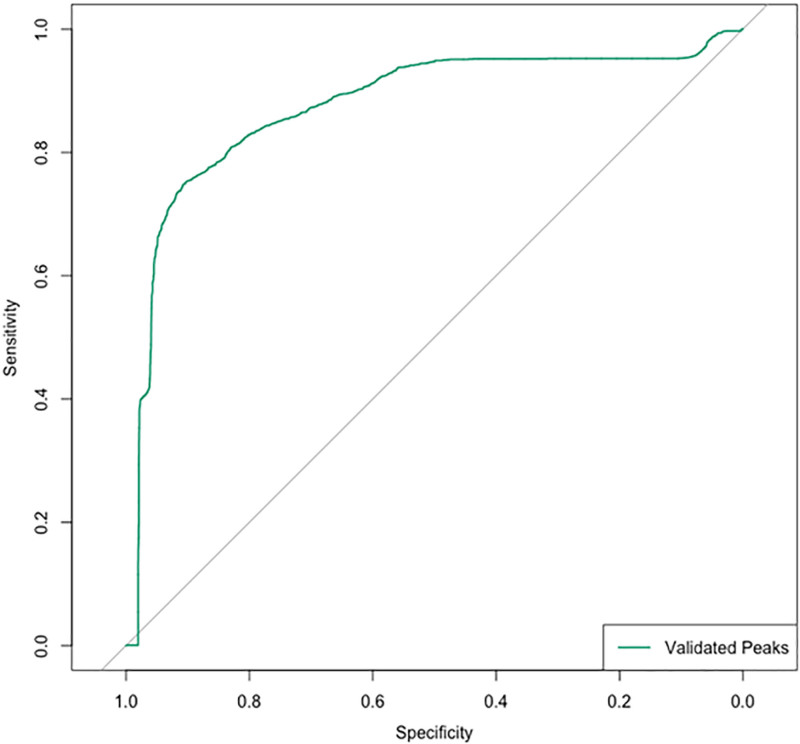
Receiver operating characteristic curve demonstrating test performance of the metabolomic panel of six validated peaks for differentiating Gram-positive and Gram-negative organisms.

Additional analysis was performed to identify metabolites capable of differentiating Gram-positive from Gram-negative infections. Seven peaks were detected corresponding to six metabolites. Betaine was increased in Gram-positive infections, while N(6)-methyllysine and four phosphatidylcholines (PCs; 36:4), PC(38:4), PC(38:6), and PC(40:6)) were elevated in Gram-negative infections (**Supplementary Table 3** and **Supplementary Figs. 7–15**, https://links.lww.com/CCX/B570). **Figure 4** depicts the ROC curve demonstrating test performance of the metabolite panel for differentiating Gram-positive and Gram-negative infections, with an AUC of 0.88 (95% CI, 0.68–1.0).

## DISCUSSION

Metabolic perturbations observed in severe infections reflect complex interplay between host gene expression, enzymatic activity, organ dysfunction, and metabolites produced by invading pathogens (23). Metabolomics, by enabling comprehensive analysis of the entire metabolome, offers a powerful platform for investigating these biochemical changes. One of the key uses of metabolomics within the ICU setting is the identification of clinically relevant biomarkers. To date, numerous studies have explored metabolomic signatures associated with primary sepsis, with applications in both diagnosis (24–30) and prognosis (31–35). Fewer studies have sought to use metabolomics to characterize different pathogens. To our knowledge, this study is the first to investigate the diagnostic utility of metabolomics for secondary infections in a critically ill COVID-19 cohort. Metabolomic profiling identified a panel of three metabolites (creatine, S-methyl-L-cysteine, and 2-hydroxyisovalerylcarnitine) that significantly differentiated patients with and without secondary infections. Additionally, six compounds (betaine, N(6)-methyllysine, PC(36:4), PC(38:4), PC(38:6), and PC(40:6)) were found to discriminate between Gram-positive and Gram-negative infections. These findings demonstrate the potential utility of metabolomics in aiding decision-making within the ICU to permit targeted antimicrobial therapy to be provided at an early opportunity. Notably, several of the identified metabolites are involved in amino acid and glycerophospholipid metabolism (**Supplementary Fig. 16**, https://links.lww.com/CCX/B570). These pathways have been shown to be dysregulated in previous sepsis metabolomic studies (25, 26, 33, 34, 36–40).

Although metabolomics datasets are generally less complex than those generated in genomics or proteomics, sepsis metabolomics studies still produce large volumes of data due to the inherent heterogeneity of infecting pathogens and the variability in host responses. The application of machine learning enables sophisticated analysis of these high-dimensional datasets. By utilizing iterative pattern recognition, artificial intelligence algorithms can continuously refine their predictive performance, improving diagnostic accuracy beyond that achievable with conventional diagnostic approaches (41, 42). Furthermore, metabolomic data can be integrated with routine clinical measurements to develop composite biomarkers of infection. For example, Zheng et al (43) employed a machine-learning model combining metabolomic features with clinical markers such as lactate, platelet count, and WCC. Their model accurately identified infected patients, achieving an AUC of 0.94. Additionally, their model could distinguish Gram-positive from Gram-negative infections with an AUC of 0.80. In our study, the metabolomic panel outperformed the traditional infection markers WCC, CRP, and procalcitonin. This may reflect redundancy, where the host inflammatory response is already captured by the metabolomic features, rendering these traditional markers superfluous. Alternatively, this may represent the limited specificity of WCC, CRP, and procalcitonin, which may be elevated in a variety of conditions, including severe COVID-19 (9).

Recent advances in metabolomic technologies have significantly improved throughput, reduced costs, and expanded the range of detectable metabolites (44). Despite these advancements, the clinical implementation of metabolomics remains constrained by the slow speed of sample processing and data analysis, as well as the complexity of result interpretation. In conditions such as secondary infections, where rapid identification is crucial for timely intervention, the current limitations of metabolomics preclude its use for real-time clinical decision-making. As such, the clinical point-of-care application of metabolomics is currently not a realistic possibility. However, faster analysis speeds and developments such as portable mass spectrometry platforms may help to address these limitations in the future (45).

Once a biomarker panel is validated with external cohorts, the ultimate aim would be to develop a clinically applicable diagnostic assay. Such an assay could permit rapid identification of secondary infections within the critically ill COVID-19 cohort, thereby supporting earlier initiation of targeted treatment (46). By bypassing the long processing times associated with mass spectrometry, a quick laboratory assay could provide a rapid result allowing for faster clinical decision-making.

While a validated biomarker panel could potentially improve diagnostic speed and accuracy, the use of a single universal panel may not adequately capture the substantial heterogeneity observed in secondary infections. Applying a one-size-fits-all approach risks overlooking important biological distinctions between different infection types. While this study sought to determine metabolomic differences between Gram-positive and Gram-negative infections, other factors such as infection source, pathogen type, and differing host immune responses may also impact the metabolome. The application of precision medicine in this context is still at an early stage of development; however, future studies aimed at identifying biomarker panels tailored to distinct patient endotypes and subphenotypes may improve the diagnostic capability of metabolomics (47, 48). A precision medicine approach could reveal novel therapeutic pathways, guiding more targeted treatment decisions. As metabolomic technologies continue to evolve, pharmacometabolomics is poised to play a key role in the management of ICU patients, enabling the customization of treatments, continuous monitoring of patient responses to therapies, and early detection of potential treatment failure (49). A recent study by Antcliffe et al (50) showed that three distinct metabolic subphenotypes exist within patients with septic shock, and persistence of the subphenotype associated with increased lipids was linked to improved survival. Serial sampling of lipid metabolites in these patients could enable the detection of those at higher risk of mortality, and therefore could trigger initiation of specific targeted metabolism-modulating therapies. In the context of secondary infections, identifying specific pathogens through metabolomic panels could allow for the early initiation of appropriate antibiotic therapy, bypassing the need to wait for the lengthy and often inconclusive results of culture and sensitivity testing.

The use of a multiomics approach may further enhance future studies within ICU by integrating complementary biological systems. This multilevel perspective could enable the characterization of both upstream regulatory mechanisms such as genetic predispositions, and downstream metabolic effects, thereby uncovering mechanistic insights into secondary infections and sepsis pathophysiology (49). This approach could generate new hypotheses and potentially identify novel drug targets.

This study has several limitations. Although the number of recruited patients exceeds that of other similar sepsis metabolomics studies, the sample size remains relatively small, which may have limited the generalizability of the findings. There was a lack of standardization due varying hospital and critical care admission times following COVID-19 onset, as well as varying secondary infection onset. The variation in timings may have prevented consistent capture of peak metabolic disturbances. This study was conducted during the early phases of the COVID-19 pandemic, when treatment strategies were evolving. All recruited patients received corticosteroids, and a proportion also received interleukin-6 or Janus kinase inhibitors. These immunomodulatory drugs may attenuate the inflammatory response observed in secondary infections, thereby decreasing the magnitude of metabolic derangement. The results presented in this study have not been validated externally. Validation using a separate external cohort is necessary before any clinical application can be considered. Crucially, this study was limited to patients with severe COVID-19, and it is unclear if this same panel would be applicable in non-COVID-19 populations. Future studies should investigate the utility of the panel in the detection of secondary infections in other forms of critical illness such as polytrauma, severe pancreatitis, or major burns.

## CONCLUSIONS

This study has identified a potential metabolomic profile for identifying secondary infections in critically ill patients with COVID-19 disease. Metabolomics, in combination with machine learning and precision medicine approaches, holds significant promise as a tool for diagnosis and management of secondary infections in critically ill patients.

## ACKNOWLEDGMENTS

We thank the three participating centers, including research staff and critical care staff, for their contribution to patient recruitment and sample collection. In particular, we thank Dr. Kathryn Puxty, Kevin Rooney, Dr. Arun Parajuli, Dr. Marcus McClean, Dr. Alex Phillips, Dr. Andrew Arnott, Dr. Maximillian Ralston, Ms. Sophie Kennedy-Hay, Mr. Steven Henderson, Ms. Susanne Cathcart, and Mr. Gary Semple. We thank Ms. Patricia Rimbi and Dr. Nicky O’Boyle for assistance with sample processing. Biorender.com was used to produce Supplementary Figure 16 (https://links.lww.com/CCX/B570). Finally, we thank all patients who volunteered to participate in this study.

## Supplementary Material
