# Supplementary material for: Metabolomics for the Diagnosis of Secondary Infections in Critically Ill Patients With COVID-19
Source: Crit Care Explor. 2025 Nov 6;7(11):e1336. doi: 10.1097/CCE.0000000000001336 (PMC12594302; doi:10.1097/CCE.0000000000001336)
Supplement: Supplementary file 1 [file cc9-7-e1336-s001.pdf]

Supplementary Digital Content

Supplementary Tables

Supplementary Table 1: Secondary infection diagnostic criteria. 2
Supplementary Table 2: Secondary infections cultured from blood, urine, respiratory tract and gastrointestinal tract 2
Supplementary Table 3: Metabolites significantly different between patients with Gram positive and Gram negative infections 4

Supplementary Figures

Supplementary Figure 1: PCA plots of the first two principal components 5
Supplementary Figure 2: Chromatogram match for creatine in pooled sample and known standard. 6
Supplementary Figure 3: MS2 fragmentation spectra match for creatine in pooled sample and known standard. 7
Supplementary Figure 4: Chromatogram match for S-methyl-L-cysteine in pooled sample and known standard. 8
Supplementary Figure 5: MS2 fragmentation spectra match for S-methyl-L-cysteine in pooled sample and known standard. 9
Supplementary Figure 6: MS2 fragmentation spectra for 2-hydroxyisovalerylcarnitine in pooled sample 10
Supplementary Figure 7: Dot plots depicting significantly different metabolites between Gram positive and negative infections: (a) betaine, (b) N(6)-methyllysine,, (c) PC(36:4), (d) PC(38:4), (e) PC(38:6), (f) PC(40:6) 11
Supplementary Figure 8: Chromatogram match for betaine in pooled sample and known standard. 12
Supplementary Figure 9: MS2 fragmentation spectra match for betaine in pooled sample and known standard. 13
Supplementary Figure 10: Chromatogram match for N(6)-methyllysine in pooled sample and known standard. 14
Supplementary Figure 11: MS2 fragmentation spectra match for N(6)-methyllysine in pooled sample and known standard. 15
Supplementary Figure 12: MS2 fragmentation spectrum for PC(36:4) 16
Supplementary Figure 13: MS2 fragmentation spectrum for PC(38:4) 17
Supplementary Figure 14: MS2 fragmentation spectrum for PC(38:6) 18
Supplementary Figure 15: MS2 fragmentation spectrum for PC(40:6) 19
Supplementary Figure 16: Arginine and glycine metabolism pathways 20

Supplementary Table 1: Secondary infection diagnostic criteria.

Infection Type	Criteria
**Bloodstream**	1) Single growth of a pathological organism<br>OR<br>2) Two or more cultures of a skin contaminant on two consecutive days.
**Respiratory Tract**	1) Growth of an organism from a BAL<br>OR<br>2) Growth of an organism from an upper respiratory tract sample<br>AND<br>Evidence of an inflammatory response (WCC >12, Temp < 36 or > 38)<br>AND<br>CXR changes<br>AND<br>O_2 requirement<br>AND<br>Purulent secretions
**Urinary Tract**	Growth of an organism with ≥ 10^5 CFU/ml<br>AND<br>Pyrexia and/or symptoms of urinary tract infection
**Gastroenteritis**	Acute onset diarrhoea >12 hours with no clear non-infectious cause<br>OR<br>At least two of the following: nausea, vomiting, abdominal pain, pyrexia or headache<br>AND<br>At least one of the following: a) Positive identification of enteric pathogen from stool or rectal swab; b) Positive identification of enteric pathogen by microscopy; c) Diagnostic single antibody titre (IgM) or 4-fold increase in paired sera (IgG) for organism.

Supplementary Table 2: Secondary infections cultured from blood, urine, respiratory tract and gastrointestinal tract. BSI: Bloodstream infection, CLABSI: Central line associated bloodstream infection

Infection	Incidence
**BSI (n=1)**	
Staphylococcus aureus	1 (100%)
**CLABSI (n=6)**	
Candida species	1 (16.6%)
Staphylococcus aureus	1 (16.6%)
Pediococcus acidilactici	1 (16.6%)
Staphylococcus hominis	1 (16.6%)
Escherichia coli	1 (16.6%)
Enterococcus faecalis	1 (16.6%)
**Urine (n=10)**	
Escherichia coli	8 (80%)
Klebsiella pneumoniae	1 (10%)
Proteus mirabilis	1 (10%)
**Chest (n=52)**	
Staphylococcus aureus	11 (21.15%)
Haemophilus influenzae	6 (11.53%)
Escherichia coli	4 (7.69%)
Klebsiella pneumoniae	4 (7.69%)
Aspergillus fumigatus	4 (7.69%)
Streptococcus pneumoniae	3 (5.77%)
Pseudomonas aeruginosa	3 (5.77%)
Proteus mirabilis	3 (5.77%)
Candida species	3 (5.77%)
Klebsiella aerogenes	2 (3.85%)
Raoultella ornithinolytica	2 (3.85%)
Enterobacter cloacae	2 (3.85%)
Raoultella planticola	1 (1.92%)
Streptococcus agalactiae	1 (1.92%)
Klebsiella oxytoca	1 (1.92%)
Stenotrophomonas maltophilia	1 (1.92%)
Morganella morganii	1 (1.92%)
**Gastrointestinal tract (n=1)**	
Campylobacter jejuni	1 (100%)
**Single infection**	21 (52.5%)
**Polymicrobial**	19 (47.5%)

Supplementary Table 3: Metabolites significantly different between patients with Gram positive and Gram negative infections

Metabolite	Direct Parent	Molecular Formula	*m/z*	Retention Time (s)	Relative Intensity (mean ± SE)		Log2 Fold Change	q-value
					Gram Positive	Gram Negative		
**Betaine**	Alpha amino acids	$C_5H_{12}NO_2$	118.0863	588	26.499 ± 0.1077	26.244 ± 0.0739	0.014	0.043
**N(6)-Methyllysine**	L-alpha-amino acids	$C_7H_{16}N_2O_2$	159.1139	1049	19.109 ± 0.1133	19.544 ± 0.1023	-0.033	0.035
**PC(36:4)**	Phosphatidylcholines	$C_{44}H_{80}NO_8P$	782.5689	178	23.615 ± 0.0907	23.971 ± 0.0508	-0.022	<0.001
**PC(38:4)**	Phosphatidylcholines	$C_{46}H_{84}NO_8P$	810.6001	179	22.668 ± 0.0928	22.896 ± 0.0476	-0.014	0.01
**PC(38:6)**	Phosphatidylcholines	$C_{46}H_{80}NO_8P$	806.5688	177	22.563 ± 0.0627	22.821 ± 0.0496	-0.016	0.009
**PC(40:6)**	Phosphatidylcholines	$C_{48}H_{84}NO_8P$	834.6000	177	20.684 ± 0.0759	20.922 ± 0.0489	-0.017	0.024

A q-value < 0.05 indicates statistical significance. SE: standard error.

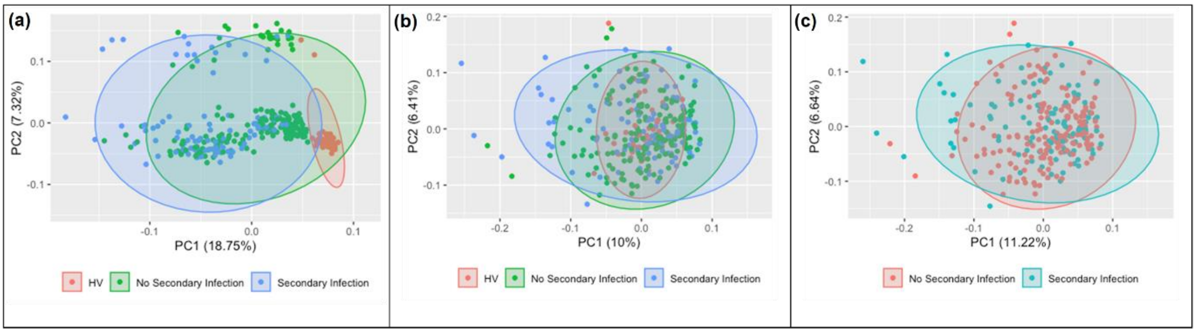

Supplementary Figure 1: PCA plots of the first two principal components: (a) full data set (b) full data set filtered (c) filtered data set with exclusion of healthy volunteers (HV)

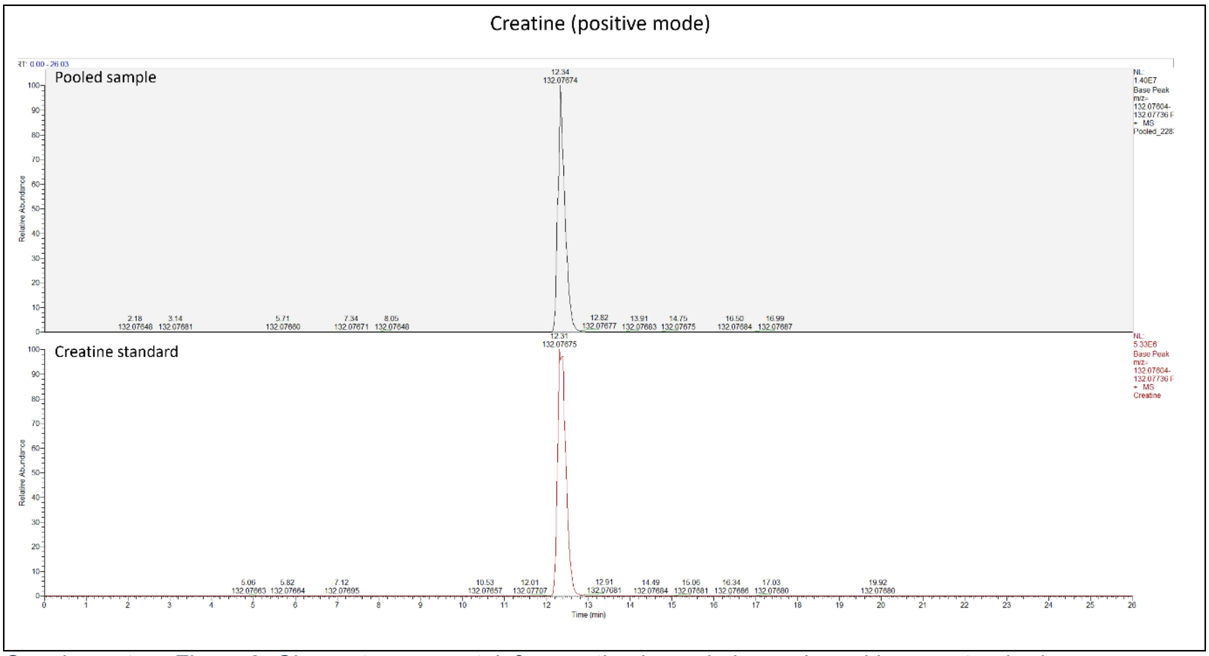

Supplementary Figure 2: Chromatogram match for creatine in pooled sample and known standard.

MS2: Creatine Peak in Sample (Positive Mode)

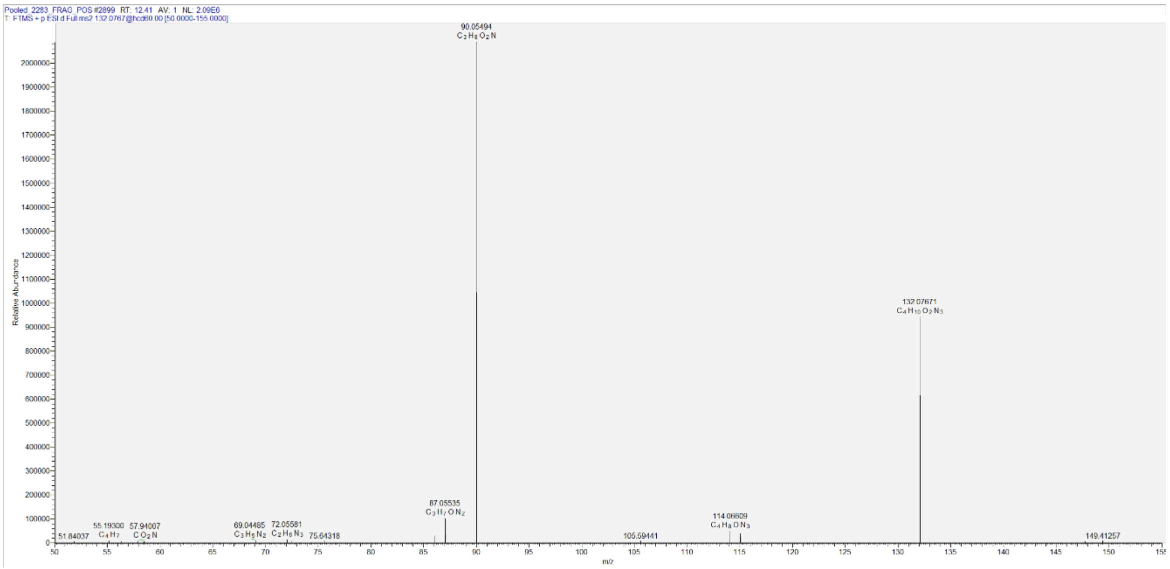

MS2: Creatine Standard Peak (Positive Mode)

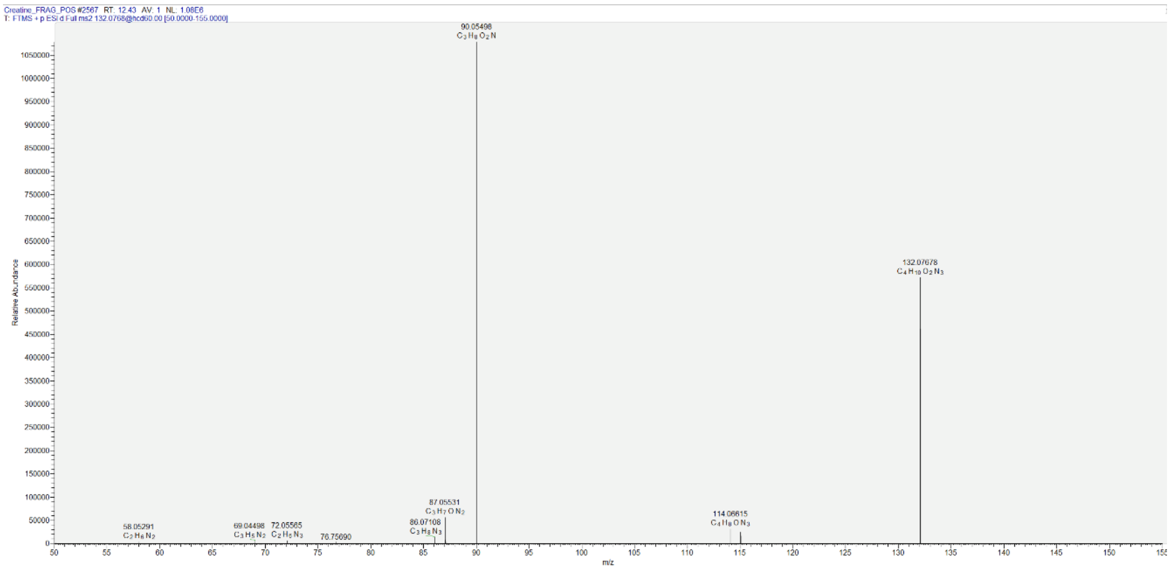

Supplementary Figure 3: MS2 fragmentation spectra match for creatine in pooled sample and known standard.

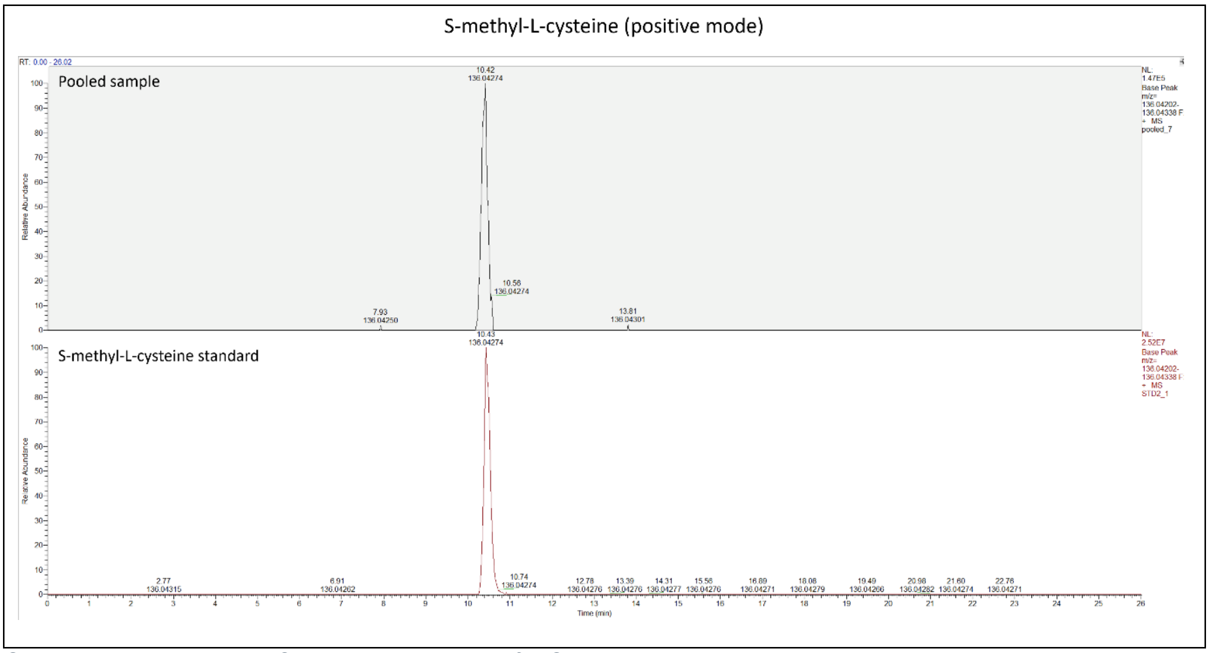

Supplementary Figure 4: Chromatogram match for S-methyl-L-cysteine in pooled sample and known standard.

MS2: S-methyl-L-cysteine Peak in Sample (Positive Mode)

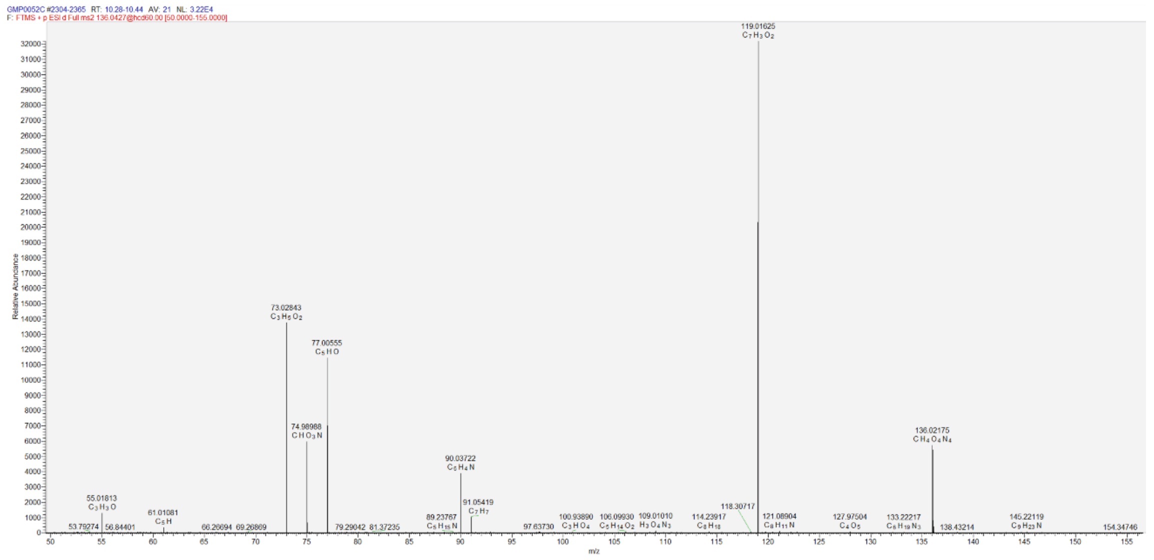

MS2: S-methyl-L-cysteine Standard Peak (Positive Mode)

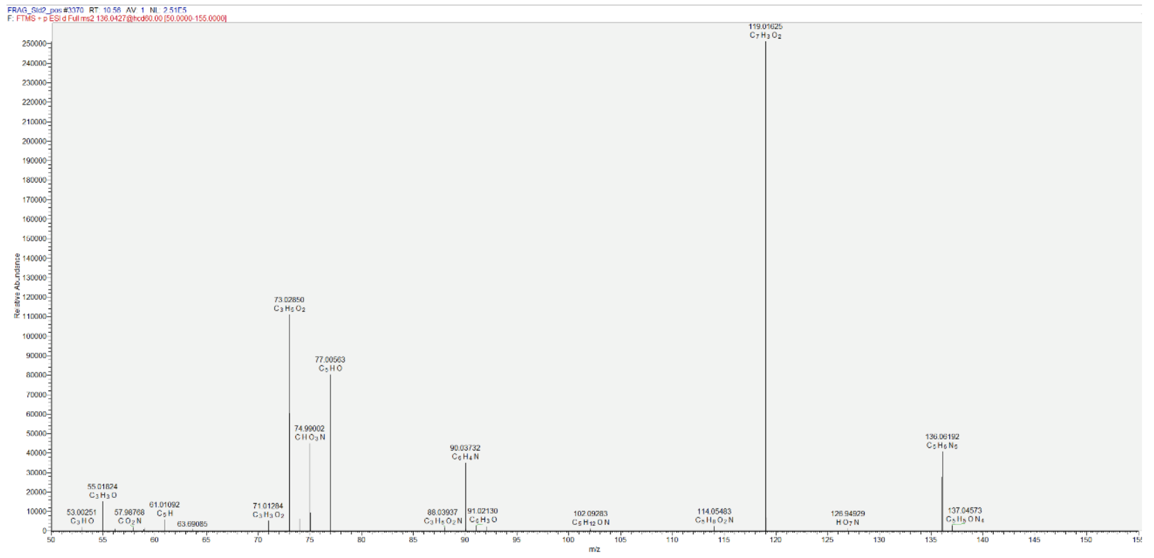

Supplementary Figure 5: MS2 fragmentation spectra match for S-methyl-L-cysteine in pooled sample and known standard.

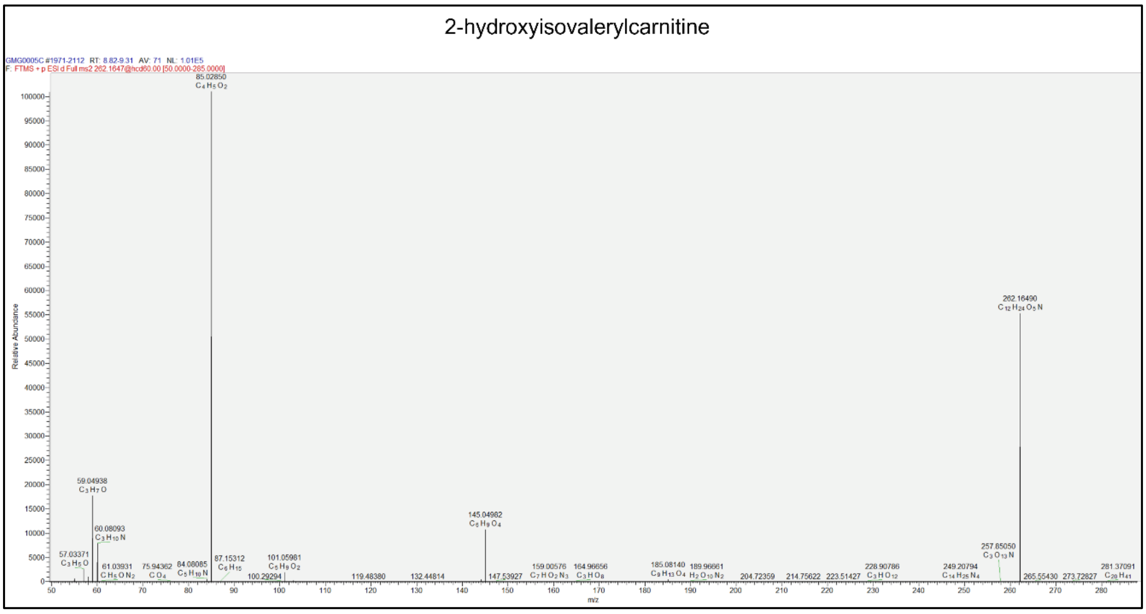

Supplementary Figure 6: MS2 fragmentation spectra for 2-hydroxyisovalerylcarnitine in pooled sample. An authentic standard for 2-hydorxyisovalerylcarnitine was unavailable for comparison, however our confidence is high in the identification of this metabolite, as the fragmentation spectrum features a fragment with m/z 85 which is known to be common amongst acylcarnitine species (1). Furthermore, the detected fragment with m/z of 145.05 is a constituent of the compound 3-hydroxyisovalerylcarnitine as reported by Maeda et.al (2).

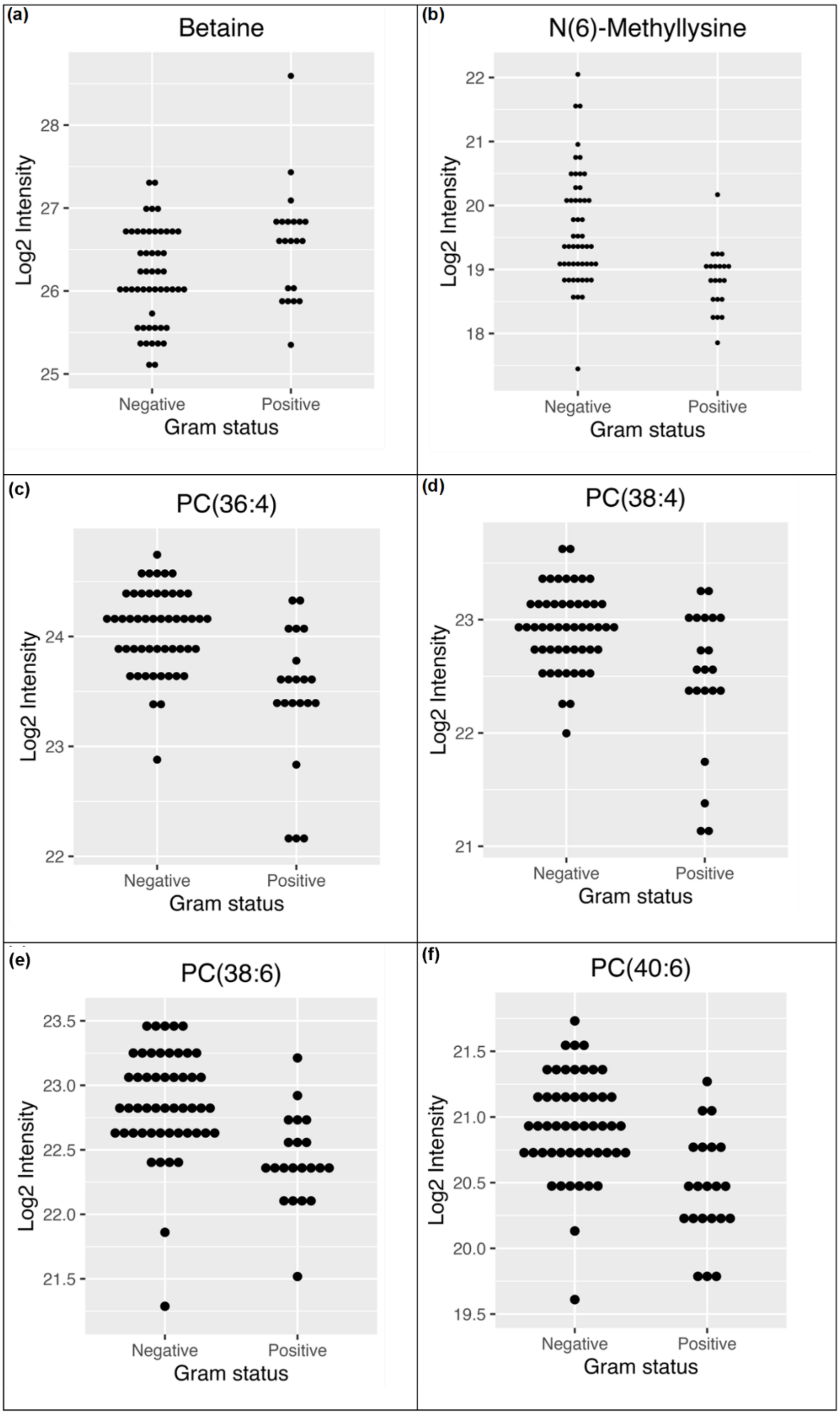

Supplementary Figure 7: Dot plots depicting significantly different metabolites between Gram positive and negative infections: (a) betaine, (b) N(6)-methyllysine,, (c) PC(36:4), (d) PC(38:4), (e) PC(38:6), (f) PC(40:6)

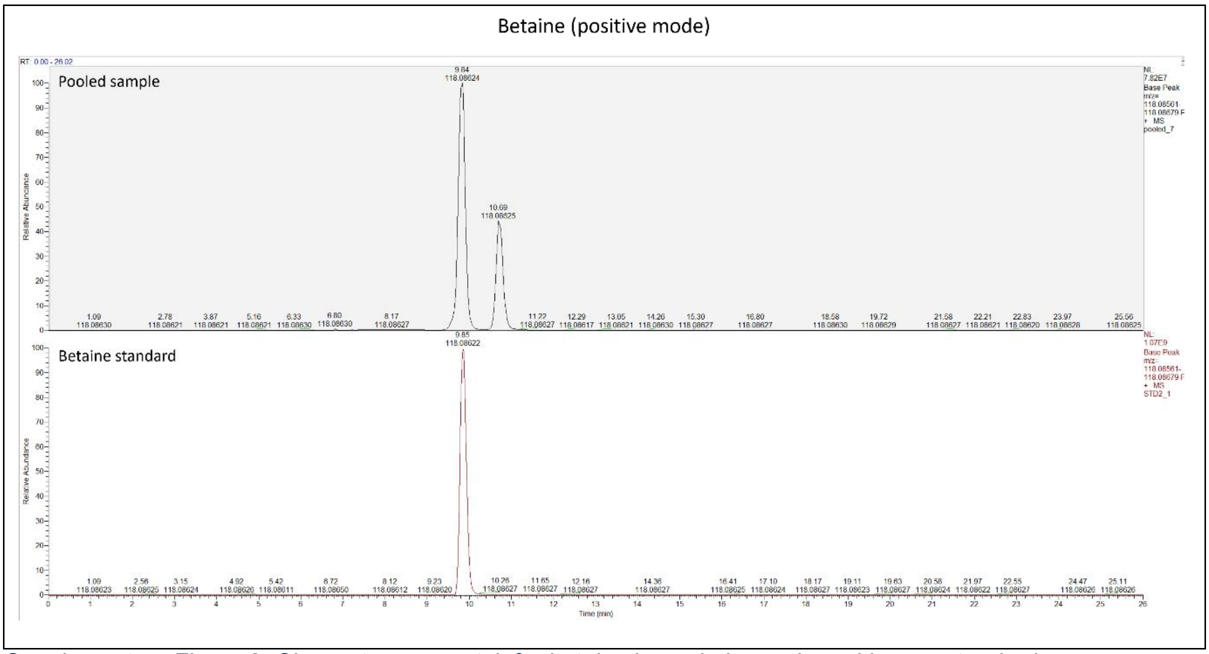

Supplementary Figure 8: Chromatogram match for betaine in pooled sample and known standard.

MS2: Betaine Peak in Sample (Positive Mode)

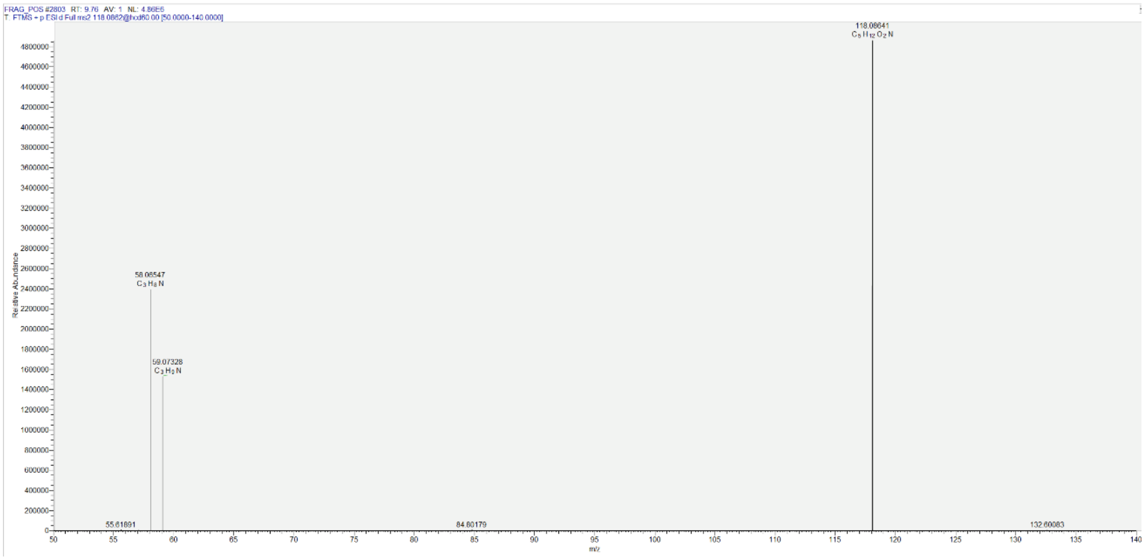

MS2: Betaine Standard Peak (Positive Mode)

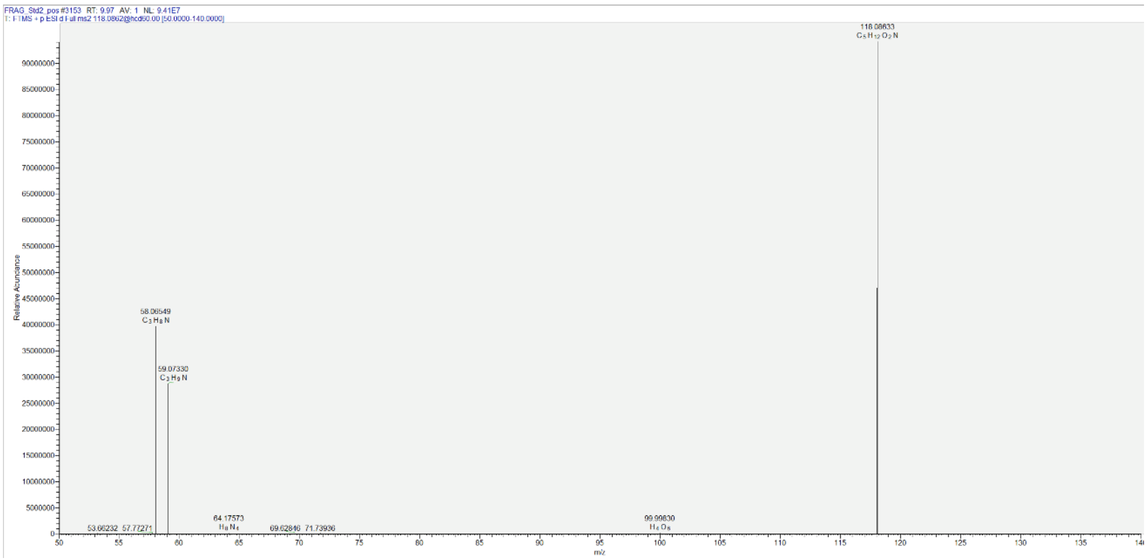

Supplementary Figure 9: MS2 fragmentation spectra match for betaine in pooled sample and known standard.

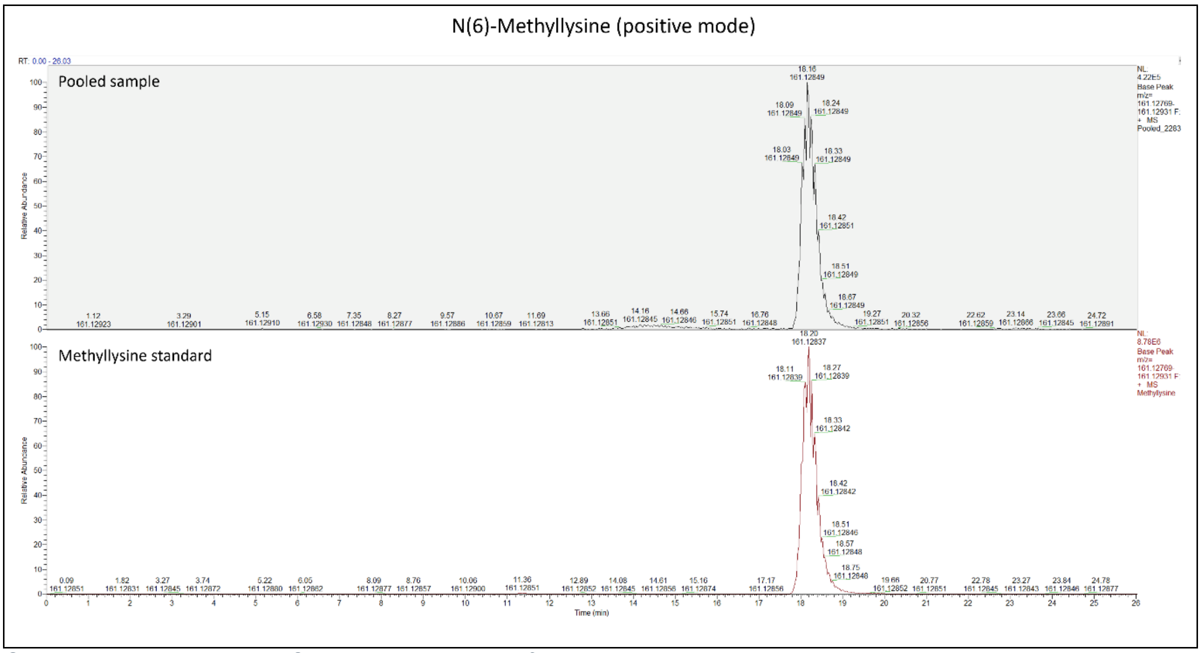

Supplementary Figure 10: Chromatogram match for N(6)-methyllysine in pooled sample and known standard.

MS2: N(6)-Methyllysine Peak in Sample (Positive Mode)

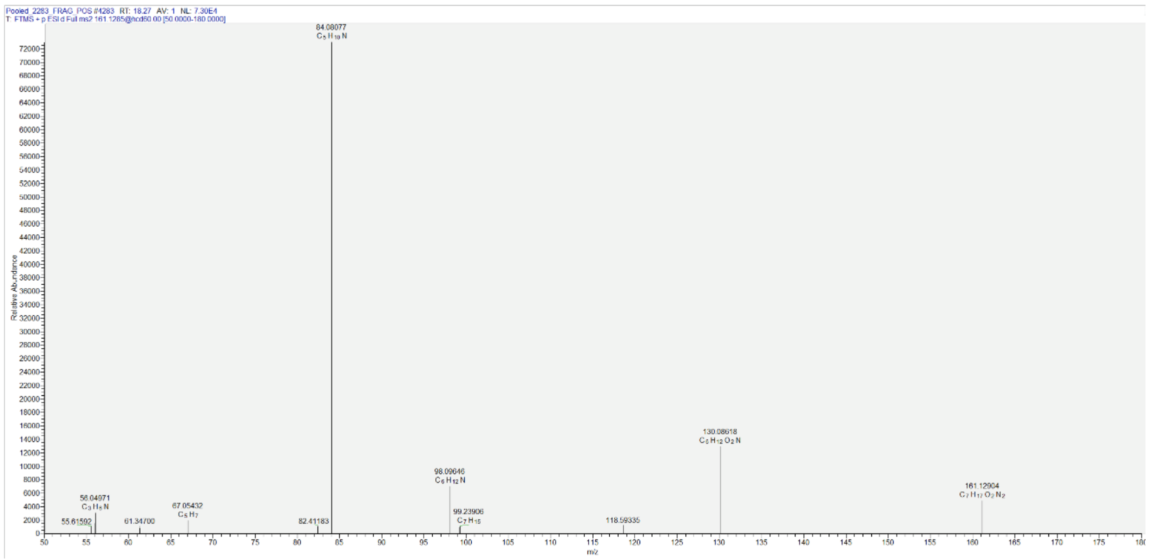

MS2: N(6)-Methyllysine Standard Peak (Positive Mode)

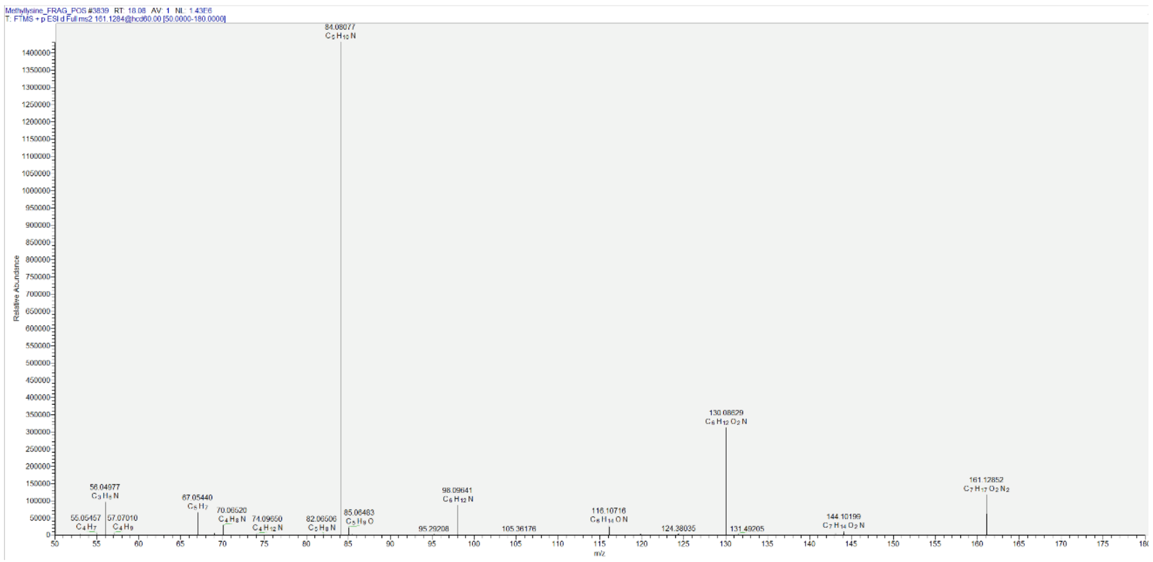

Supplementary Figure 11: MS2 fragmentation spectra match for N(6)-methyllysine in pooled sample and known standard.

Authentic standards were unavailable for the phosphatidylcholine metabolites. However, each fragmentation spectrum contains characteristic fragments of phosphatidylcholine heads with m/z 60.08108, 71.0731, 86.0965, 98.9843, 125.0000 and 184.0736.

MS2: PC(36:4) (positive mode)

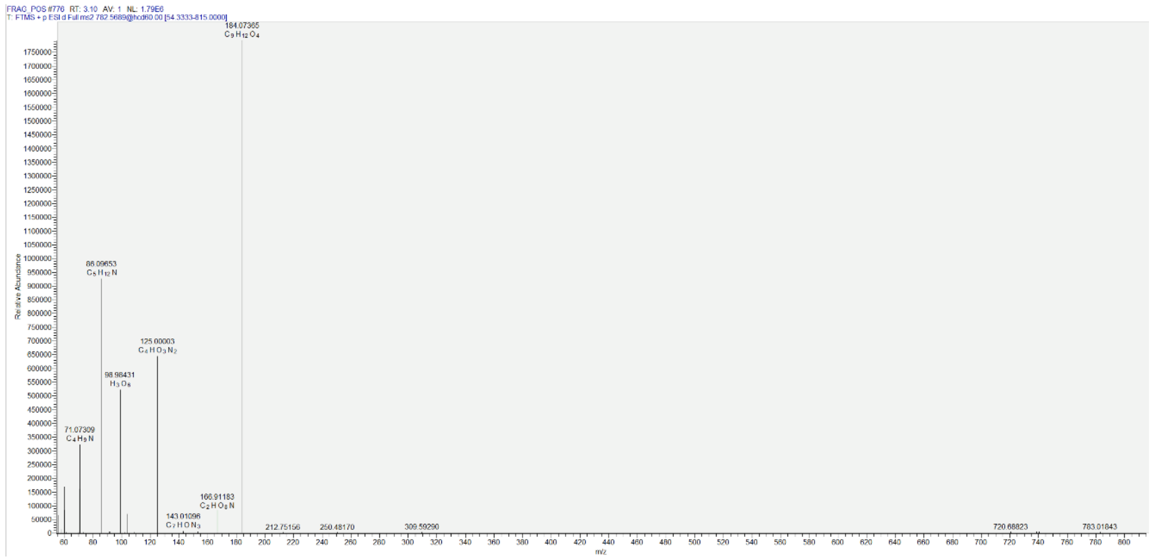

Supplementary Figure 12: MS2 fragmentation spectrum for PC(36:4)

MS2: PC(38:4) (positive mode)

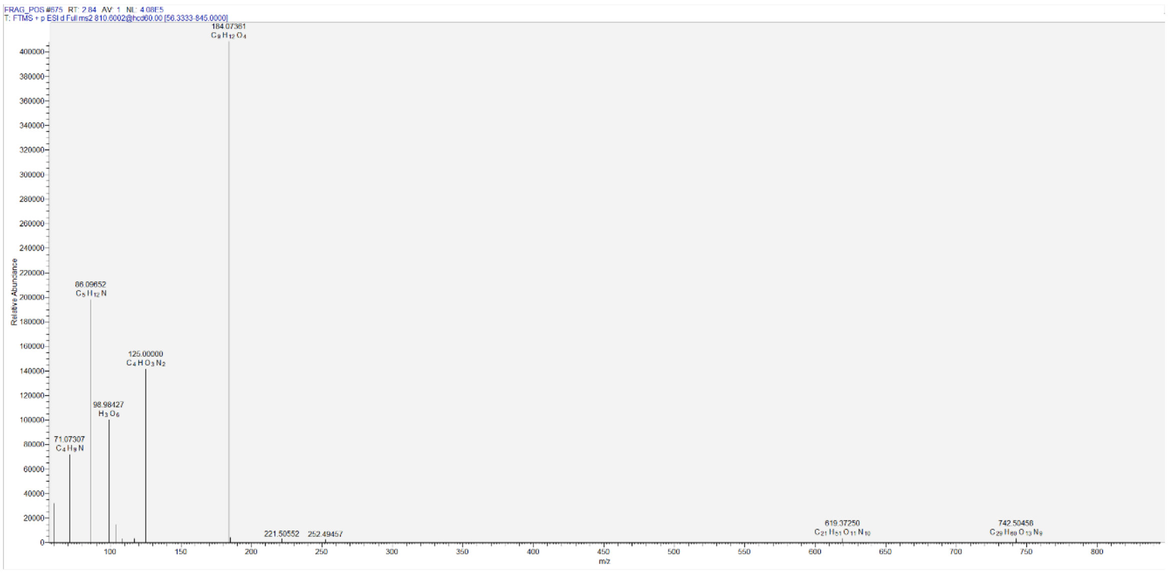

Supplementary Figure 13: MS2 fragmentation spectrum for PC(38:4)

MS2: PC(38:6) (positive mode)

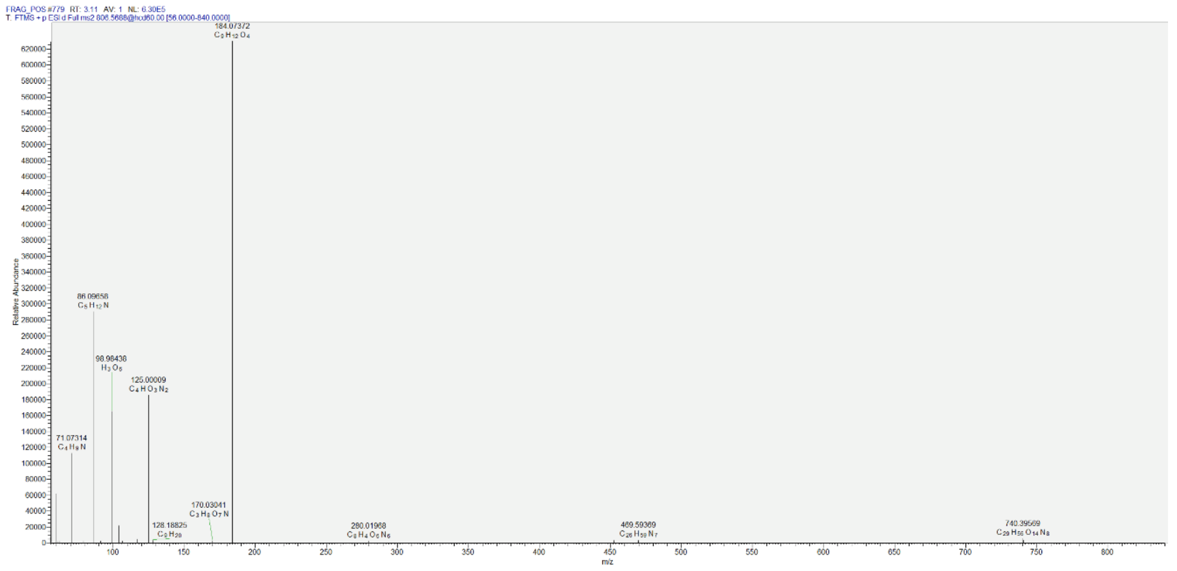

Supplementary Figure 14: MS2 fragmentation spectrum for PC(38:6)

MS2: PC(40:6) (positive mode)

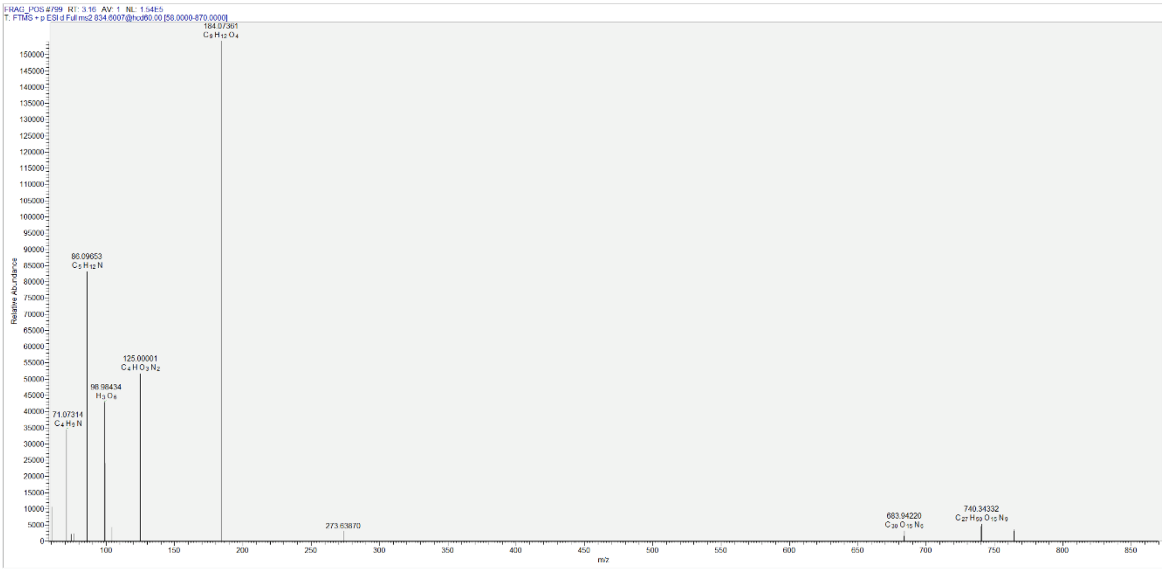

Supplementary Figure 15: MS2 fragmentation spectrum for PC(40:6)

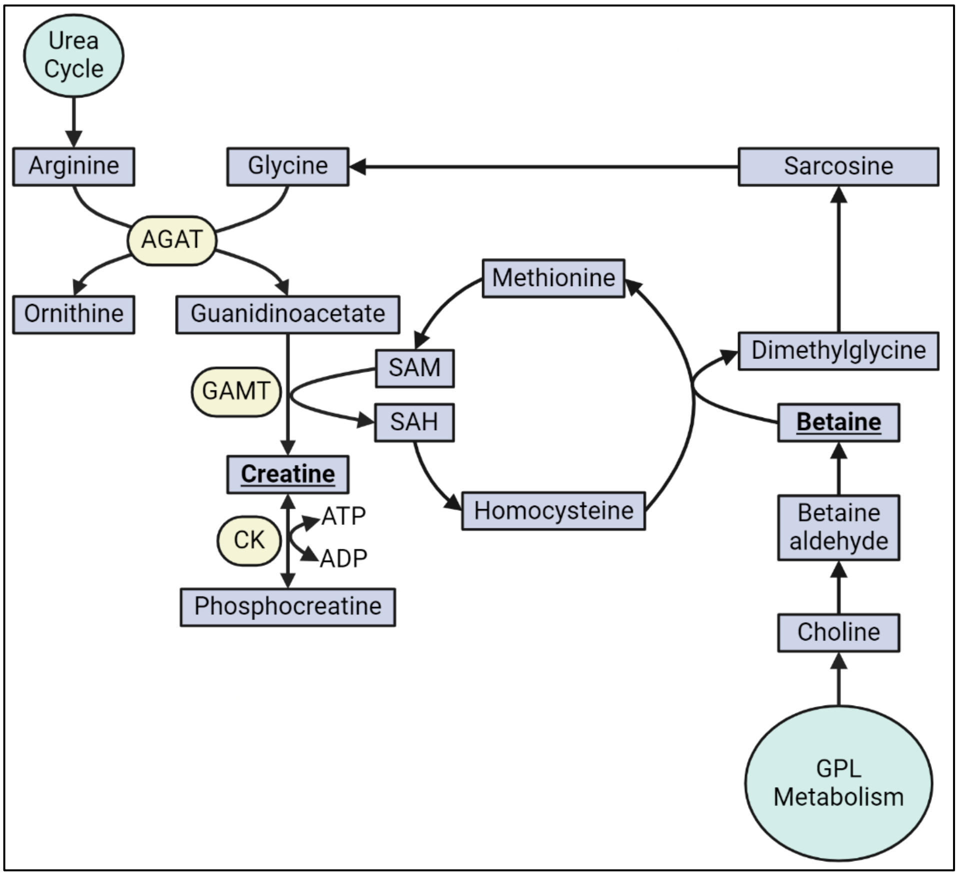

Supplementary Figure 16: Arginine and glycine metabolism pathways. Adapted from Andrade (3). ADP: Adenosine diphosphate; AGAT: Arginine:glycine amidinotransferase; ATP: Adenosine triphosphate; CK: Creatine kinase; GAMT: Guanidinoacetate N-methyltransferase; GPL: Glycerophospholipid; SAH: S-adenosylhomocysteine; SAM: S-adenosylmethionine. Figure produced using biorender, available from https://BioRender.com/z88e415

References

1. Chace DH, Hillman SL, Van Hove JL, Naylor EW. Rapid diagnosis of MCAD deficiency: quantitative analysis of octanoylcarnitine and other acylcarnitines in newborn blood spots by tandem mass spectrometry. Clin Chem. 1997 Nov;43(11):2106–13.
2. Maeda Y, Ito T, Ohmi H, Yokoi K, Nakajima Y, Ueta A, et al. Determination of 3-hydroxyisovalerylcarnitine and other acylcarnitine levels using liquid chromatography-tandem mass spectrometry in serum and urine of a patient with multiple carboxylase deficiency. J Chromatogr B, Anal Technol Biomed life Sci. 2008 Jul;870(2):154–9.
3. Andrade F. The arginine-creatine pathway is disturbed in children and adolescents with renal transplants (Pediatric Research (2008) 64 (218-222). Pediatr Res. 2009;65(2):248.
